# Gestational weight gain according to pre-pregnancy body mass index of a group of Latin American adolescents and its association with newborn birth weight

**DOI:** 10.1590/0102-311XEN130524

**Published:** 2025-11-10

**Authors:** Keren Cano-Pulgarín, Alejandro Estrada-Restrepo, Josué Cano, Odalis Sinisterra, Cecilia Severi, María del Carmen Zimmer-Sarmiento, Reyna Sámano, María Victoria Benjumea-Rincón, Maria Isabel López-Ocampos, Sandra Lucía Restrepo-Mesa

**Affiliations:** 1 Universidad de Antioquia, Medellín, Colombia.; 2 Ministerio de Salud de la República de Panamá, Ancón, Panamá.; 3 Facultad de Medicina, Universidad de la República, Montevideo, Uruguay.; 4 Centro Latinoamericano de Perinatología, Salud de la Mujer y Reproductiva, Montevideo, Uruguay.; 5 Universidad Nacional de Salta, Salta, Argentina.; 6 Instituto Nacional de Perinatología de México, Ciudad de México, México.; 7 Universidad María Auxiliadora, Mariano Roque Alonso, Paraguay.

**Keywords:** Pregnancy in Adolescence, Gestational Gain Weight, Body Mass Index, Birth Weight, Embarazo en Adolescencia, Ganancia de Peso Gestacional, Índice de Masa Corporal, Peso al Nacer, Gravidez na Adolescência, Ganho de Peso na Gestação, Índice de Massa Corporal, Peso ao Nascer

## Abstract

This study aims to analyze the distribution of gestational weight gain in a group of Latin American adolescents according to their pre-pregnancy body mass index (BMI, based on the World Health Organization criteria for adolescents and adults) and its association with their newborns’ birth weight. This longitudinal retrospective study used secondary data from national or institutional perinatal information systems about pregnant adolescents from Argentina, Colombia, Mexico, Panama, Paraguay, and Uruguay. The degree of agreement between the two classification criteria for the pre-pregnancy BMI was determined with the B statistic and the Bangdiwala graph. The association of newborns’ weight with the pre-pregnancy BMI and the gestational weight gain was assessed using regression models. This study included 6,141 pregnant adolescents. When compared to the adolescents’ criterion, the pre-pregnancy BMI classification for adults tends to underestimate the assigned category, leading to a higher recommended weight gain. Regardless of the criterion, overweight and high gestational weight gain were significantly associated with a higher probability of newborns with macrosomia and birth weight > P90, obesity was associated with birth weight > P90, and low weight gain was associated with low, insufficient, and < P10 birth weight. In conclusion, pre-pregnancy BMI and gestational weight gain are associated with the birth weight of newborns from Latin American adolescents.

## Abstract (es)

El objetivo de este artículo es analizar la distribución del aumento de peso gestacional de un grupo de adolescentes latinoamericanas según su índice de masa corporal (IMC) previo al embarazo, clasificado según los criterios de la Organización Mundial de la Salud para adolescentes y adultos, y su asociación con el peso del recién nacido al nacer. Este estudio longitudinal y retrospectivo utiliza datos secundarios de sistemas de información perinatal nacionales o institucionales sobre adolescentes embarazadas de Argentina, Colombia, México, Panamá, Paraguay y Uruguay. El grado de concordancia entre los dos criterios de clasificación del IMC previo al embarazo se determinó con la estadística B y el gráfico de Bangdiwala. La asociación del peso del recién nacido tanto con el IMC pregestacional como con la ganancia de peso gestacional se evaluó mediante modelos de regresión. El estudio incluyó a 6.141 adolescentes embarazadas. En comparación con el criterio para adolescentes, el criterio de clasificación del IMC previo al embarazo para adultos tiende a subestimar la categoría asignada, lo que lleva a un aumento de peso recomendado más alto. Independientemente del criterio utilizado, el sobrepeso y el aumento de peso gestacional elevado se asociaron significativamente con una mayor probabilidad de que los recién nacidos presentaran macrosomía y un peso al nacer > P90, la obesidad se asoció con un peso al nacer > P90, además, el bajo aumento de peso se asoció con un peso al nacer bajo, insuficiente y < P10. En conclusión, el IMC previo al embarazo y el aumento de peso gestacional se asocian con el peso de los recién nacidos al nacer de las adolescentes latinoamericanas.

## Abstract (pt)

Este artigo tem como objetivo analisar a distribuição do ganho de peso gestacional em um grupo de adolescentes latino-americanas de acordo com seu índice de massa corporal (IMC) pré-gestacional, classificado com base nos critérios da Organização Mundial da Saúde para adolescentes e adultas, e sua associação com o peso ao nascer do recém-nascido. Trata-se de um estudo longitudinal e retrospectivo que utilizou dados secundários de sistemas de informação perinatal nacionais ou institucionais sobre adolescentes grávidas de seis países da América Latina: Argentina, Colômbia, México, Panamá, Paraguai e Uruguai. O grau de concordância entre os dois critérios de classificação do IMC pré-gestacional foi determinado com a estatística B e o gráfico de Bangdiwala. As associações do peso do recém-nascido com o IMC pré-gestacional e o ganho de peso gestacional foram avaliadas usando modelos de regressão. O estudo teve a participação de 6.141 adolescentes grávidas. Comparado ao critério para adolescentes, o critério de classificação do IMC pré-gestacional para adultos tende a subestimar a categoria atribuída, levando a um maior ganho de peso recomendado. Independentemente do critério utilizado, sobrepeso e alto ganho de peso gestacional associaram-se significativamente a uma maior probabilidade de recém-nascidos com macrossomia e peso ao nascer > P90; obesidade estava associada com peso ao nascer > P90; e baixo ganho de peso estava associado com peso ao nascer baixo, insuficiente e < P10. Em conclusão, o IMC pré-gestacional e o ganho de peso gestacional estão associados ao peso ao nascer de recém-nascidos de adolescentes latino-americanas.

## Introduction

Adolescent pregnancy significantly increases health risks for the mother-child dyad. Pregnancy and childbirth complications constitute the second leading cause of death in women aged 15 to 19 years worldwide. Their children face a two to three times higher risk of mortality than newborns of adult women ^1^ and more frequent adverse outcomes, such as prematurity, low birth weight, and small for gestational age newborns ^2^ ^,^ ^3^. 

Despite its serious consequences, adolescent pregnancy numbers remain alarming in Latin America: the second highest rate in the world (66.5/1,000 births). Besides, it has the slowest decline in the 15 to 19 age group, and it is the only region with an upward trend in girls aged under 15 years ^4^.

Moreover, newborn weight - considered the primary neonatal indicator due to its impact on health and nutrition throughout life ^5^ - suffers the influence of pre-pregnancy body mass index (BMI) and maternal weight gain ^6^ ^,^ ^7^ ^,^ ^8^, accentuating the importance of adequately evaluating these indicators in adolescent pregnant women. However, there is still no consensus on the appropriate criterion to classify pre-pregnancy BMI nor specific weight gain recommendations for this group.

In 2009, the Institute of Medicine (IoM) of the United States ^9^ published weight gain recommendations for adult pregnant women but cited insufficient evidence for adolescents. Although adolescent-specific BMI classification criteria exist ^10^, the IoM recommended using the adult World Health Organization (WHO) criterion ^11^. Studies ^12^ ^,^ ^13^ ^,^ ^14^ have shown discrepancies between these criteria, which may lead to inappropriate weight gain recommendations and adverse effects on maternal and newborn health.

According to our knowledge, no study has addressed the behavior of pre-pregnancy BMI and the weight gain of adolescent mothers in association with the birth weight of their children in Latin America. That information is essential to design timely, specific, and contextualized nutritional surveillance strategies to improve the development of the region and the health and nutrition of the adolescent mother-child dyad.

Thus, this study aimed to analyze the distribution of gestational weight gain in Latin American adolescents according to their pre-pregnancy BMI (according to the WHO criteria for adolescents and adults) and their association with newborn birth weight.

## Materials and methods

### Study design

An observational, longitudinal, retrospective study was carried out based on 25,815 records from databases of adolescent pregnant women and their newborns from Argentina, Colombia, Panama, Paraguay, Mexico, and Uruguay. The databases (using national or institutional perinatal information systems compiled from 2008 to 2019) were provided by researchers and officials from health ministries or institutions. A standardized form was used to collect the necessary data from each study and assess their eligibility for inclusion in the combined dataset. The forms were reviewed by the project core research team. Datasets from studies containing the required variables were subsequently requested.

**Figure 1:**
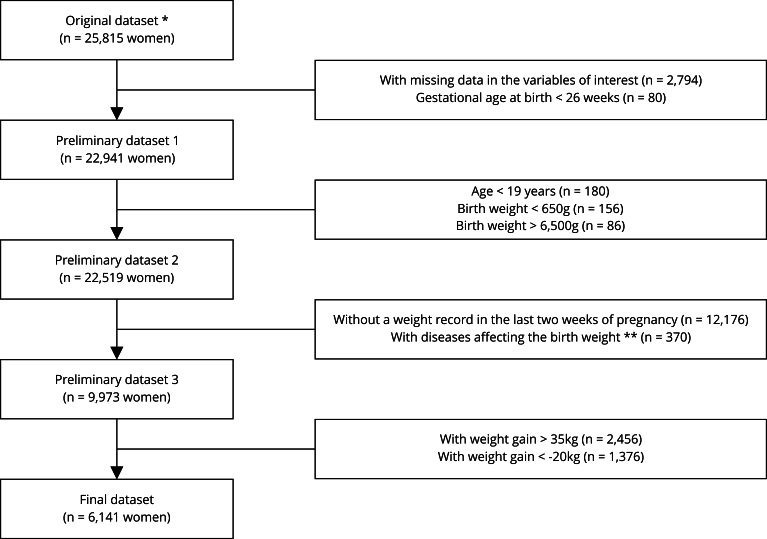
Data depuration process for databases of Latin American pregnant women and their newborns.

Each invited researcher provided the following details about their studies: the origin of the study, municipality and country of data collection, data source (primary or secondary), dataset sample size, maternal age/date of birth, presence of obstetric and previous diseases (such as diabetes mellitus, hypertension), pregestational weight and height, weight at the end of pregnancy, gestational age at birth, and newborns’ sex and birth weight. All selected datasets were individually and carefully revised during the data-cleaning process. The inclusion criteria were: woman aged from 10 to 19 years with pre-pregnancy weight data and a weight record taken in the last two weeks of pregnancy; total weight gain from -20kg to 35kg; no report in the database of diseases during pregnancy diseases that could affect the maternal weight gain and the birth weight of the newborn (such as hypertension, preeclampsia, gestational diabetes, tuberculosis, or cardiovascular diseases); ≥ 26 week gestational age at birth; delivery of a live born singleton infant; birth weight from 650g to 6,500g; no report of genetic syndromes and fetal malformations. Records of pregnant women with incomplete variables of interest (maternal age, pre-pregnancy weight and height, pre-pregnancy BMI, height-for-age, gestational weight gain, gestational age at birth, and newborns’ sex and birth weight) were excluded from this study. Finally, 6,141 dyads of adolescent pregnant women and their newborns were included (Figure 1).

* The original dataset included cases of live born singleton deliveries with available pregestational weight data; ** Diseases considered: chronic hypertension or hypertensive disorders during pregnancy, diabetes mellitus or gestational diabetes, tuberculosis, or cardiovascular diseases.

### Maternal age

Maternal age (in years) was calculated as the difference between the pregnant individual’s date of birth and the estimated date of conception. The date of conception was determined using the algorithm provided by the “ob Wheel” tool (https://obwheel.quartertone.net/).

Girls aged from 10 to 14 years were considered as early adolescents, whereas those, from 15 to 19 years, as late adolescents ^15^.

### Pre-pregnancy BMI classification

Pre-pregnancy weight and height were used to calculate pre-pregnancy BMI (kg/m^2^). Pre-pregnancy weight was collected from either of three sources: direct measurement, abstraction from medical records, or self-report. Height was measured either at the time of study enrollment or at the beginning of prenatal care. Adolescents’ pre-pregnancy BMI was classified under two WHO criteria: (1) adolescent criterion, using standard deviations in growth charts for BMI by sex and age ^16^: thinness < -2 standard deviations (SD); adequate from -2 to +1 SD; overweight > +1 and ≤ +2 SD; obesity: > +2 SD; (2) adult criterion ^17^: thinness < 18.5kg/m^2^; adequate ≥ 18.5 and < 25.0kg/m^2^; overweight from 25.0 to 29.9kg/m^2^; obesity ≥ 30kg/m^2^.

### Gestational weight gain

According to the information available in the database, weight gain was differently calculated and classified for women with full-term and preterm newborns. The total weight gain in kilograms was established as the difference between the mother’s weight in the last two weeks of pregnancy and their pre-pregnancy weight. The weekly weight gain gross rate in kilograms was calculated by dividing the total weight gain by the number of pregnancy weeks. To consider the total duration of pregnancy in adolescents with full-term newborns, the total weight gain was adjusted by adding the weight gain rate corresponding to the number of weeks of difference between the birth of the child and the last weight measurement of the mother.

According to pre-pregnancy BMI - which was obtained with two classification criteria (for adults and adolescents), weight gain was classified as insufficient, adequate, or excessive when compared with the IoM recommendations ^9^. The following recommendations were taken as a reference for total weight gain in women with full-term newborns: low weight (from 12.5 to 18kg); adequate (from 11.5 to 16kg); overweight (from 7 to 11.5kg); and obesity (from 5 to 9kg). For women with pre-term newborns, the recommended weight gain per week were used as references: low weight (from 0.44 to 0.58kg); adequate pre-pregnancy BMI (from 0.35 to 0.50kg); overweight (from 0.23 to 0.33kg); and obesity (from 0.17 to 0.27kg).

### Gestational age

Gestational age in each visit was available in the datasets and was not recalculated. Newborns delivered at 37 weeks of gestation or later were categorized as full-term, whereas those born before 37 weeks, as preterm. The databases from Mexico and Argentina included no records of preterm newborns.

### Birth weight

Birth weight was available in each dataset, and it was not possible to know its origin (if it was measured in the study, obtained from medical records, or reported by the mother). For all newborns, birth weight percentiles were calculated by sex and gestational age using the on-line tool of the International Fetal and Newborn Growth Consortium for the 21st Century (INTERGROWTH 21st) ^18^ ^,^ ^19^ ^,^ ^20^. The newborns were classified into the following categories: < percentile 3 (P3), < P10, from P10 to P90, and > P90. For full-term newborns, birth weight was also evaluated and classified as: low (< 2,500g) ^21^; insufficient (from 2,500 to 2,999g) ^22^ ^,^ ^23^ ^,^ ^24^; adequate (from 3,000 to 4,000g) ^22^ ^,^ ^23^ ^,^ ^24^; and macrosomia (> 4,000g) ^25^.

### Ethical considerations

This study was approved by the bioethics committee of the Faculty of Dentistry at Antioquia University in act 1, February 11, 2021 (concept 68-2021). The guidelines set out in the *Nuremberg Code* ^26^ and the *Helsinki Declaration* ^27^ were followed. A confidentiality agreement was signed by each investigator. Only de-identified data were used in the analyses, which were performed by authorized investigators. The main investigator of Universidad de Antioquia signed a confidentiality and custody agreement for the data with the investigator or institutional representative of each country.

### Statistical analysis

The demographic and anthropometric continuous variables were described by estimating arithmetic means, standard deviations, medians, and interquartile ranges. Proportions were calculated for categorical variables and 95% confidence intervals (95%CI) were estimated. The assumption of normality of the numerical variables was verified using the Kolmogorov-Smirnov test. The Kruskal-Wallis test was applied to compare each variable across countries.

The heterogeneity of height-for-age z scores according to pre-pregnancy BMI across the six countries in this study was assessed by standardized site differences ^28^. According to Cohen ^29^, differences of 0.2 SD units are considered small; 0.5 SD ones, acceptable; and 0.8 SD are large. Thus, standardized site differences within the range of ±0.5 units were considered homogeneous. The agreement between the two WHO criteria for the classification of the pre-pregnancy BMI and the weight gain was calculated by the McNemar’s change test and the weighted Cohen’s kappa coefficient. To expand the analysis of the classification of pre-pregnancy BMI, the Bangdiwala statistic and chart ^30^ were also calculated, evaluating the degree of agreement for each category of pre-pregnancy BMI. The Bangdiwala B statistic evaluates agreement between two methods for ordinal categorical data. Its chart complements kappa and similar statistics by visually highlighting disagreement patterns. In a k × k contingency table, rectangle areas represent marginal frequencies, whereas shaded squares on the diagonal indicate agreement. Perfect agreement emerges as fully shaded squares ^30^. The Spearman’s correlation test was used to conduct a bivariate analysis between the classification of weight gain and the classification of pre-pregnancy BMI according to the two WHO criteria; this analysis was additionally performed by age group (early and late adolescents).

For full-term newborns, a multivariate multinomial logistic regression was conducted to determine the association between pre-pregnancy BMI, gestational weight gain, and newborn outcomes. The model included pre-pregnancy BMI and gestational weight gain that were adjusted for country, height, and age and estimated odds ratios (OR) with 95%CI.

For preterm newborns, a multiple linear regression was performed following a Box-Cox transformation to birth weight. This model also included pre-pregnancy BMI and gestational weight gain, adjusted for country, height, and age.

Statistical analyses were performed on IBM SPSS version 26.0.0.0 (https://www.ibm.com/). The Bangdiwala agreement charts were created using the *VCD* package on R (http://www.r-project.org). P-values < 0.05 were considered statistically significant.

## Results

### Population characteristics

The highest percentage of data came from Panama (47.2%), followed by Uruguay (36.5%), Argentina (9%), Mexico (5%), Colombia (1.6%), and Paraguay (0.7%). Most girls were aged from 15 to 19 years, averaging 17.1±1.6 years. According to the INTERGROWTH-21st percentiles, about 10% of newborns had a birth weight for gestational age above the 90th percentile; for full-term infants, when birth weight was assessed in grams, insufficient weight configured the most frequent alteration (Table 1). Countries differed for age, weight, height, and pre-pregnancy BMI (p < 0.01) (data not shown).

**Table 1:** Population characteristics.

Parameter	Mean (SD)	Median (P25; P75)
Age (years)	17.1 (1.6)	17.0 (16.0; 18.0)
Pre-pregnancy weight (kg)	55.2 (10.0)	54.0 (49.0; 60.0)
Height (cm)	156.1 (6.6)	156.0 (152.0; 160.0)
Pre-pregnancy BMI (kg/m^2^)	22.7 (3.9)	22.1 (19.8; 24.8)
Gestational age at birth (weeks)	38.8 (1.5)	39.0 (38.0; 40.0)
Birth weight (g)	3,169.5 (460.3)	3,180.0 (2,900.0; 3,460.0)
	n	%
Maternal age (years)		
Early adolescents (10-14)	419	6.8
Late adolescents (15-19)	5,722	93.2
Sex of the newborn		
Male	3,134	51.0
Female	3,007	49.0
Classification according to gestational age at birth		
Full-term newborns	5,842	95.1
Pre-term newborns	299	4.9
Birth weight: full-term newborns (n = 5,842)		
Low weight	211	3.6
Insufficient	1,485	25.4
Adequate	3,988	68.3
Macrosomia	158	2.7
Weight for gestational age at birth: full-term newborns (n = 5,842)		
< P3	96	1.6
< P10	309	5.3
P10 to P90	4,877	83.5
> P90	560	9.6
Weight for gestational age at birth: pre-term newborns (n = 299)		
< P3	11	3.7
< P10	15	5.0
P10 to P90	235	78.6
> P90	38	12.7

BMI: body mass index; P: percentile; SD: standard deviation.

This study observed no heterogeneity in height-for-age z-scores, with most standardized site differences for both indicators ranging from -0.25 and +0.25. Thus, we considered adolescents from all countries as a single group.

### Pre-pregnancy BMI classification

When comparing pre-pregnancy BMI categories, the WHO adolescent criterion found fewer cases of thinness than the adult criterion (1.5% vs. 11.3%) but more cases of adequate weight, overweight, and obesity (adolescent criterion: 69.8%, 21.9%, and 6.8%; adult criterion: 65.2%, 18.3%, and 5.2%). The McNemar’s test showed significant changes (p < 0.01), with moderate agreement between criteria (Cohen’s kappa: 0.68, p < 0.001).

**Figure 2:**
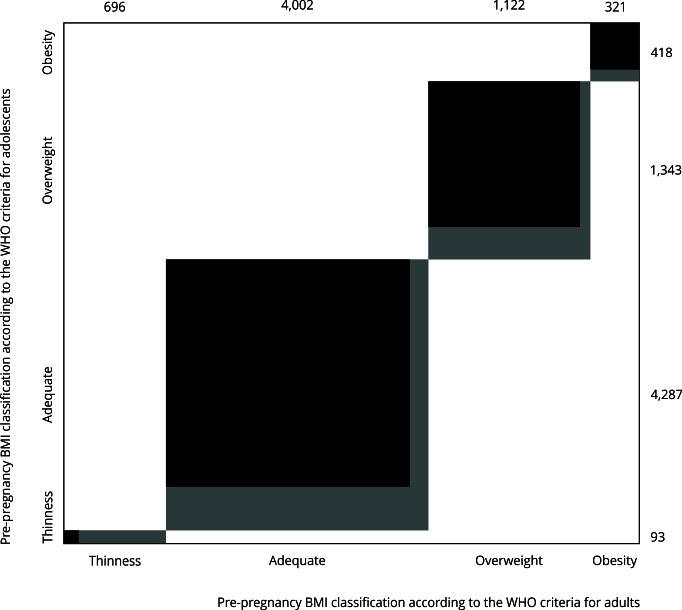
Degree of agreement between the pre-pregnancy body mass index (BMI) classifications according to the World Health Organization (WHO) criteria for adolescents and adults.

This study found a high degree of agreement between the two pre-pregnancy BMI classification criteria (statistical B = 0.98), with a tendency of the WHO criterion for adults to underestimate the resulting category when compared to the adolescent criterion. The category of thinness showed the lowest agreement and the category of obesity, the highest one (Figure 2). 

The degree of agreement increased with age, and was almost perfect for 19-year-olds in the overweight and obesity categories.

### Gestational weight gain

According to the information available in the database, the women who gave birth to full-term newborns showed a 11.9±6.6kg mean total weight gain, whereas those who conceived pre-term newborns, a 0.28±0.20kg/week mean weight gain rate. The proportion of pregnant women in each weight gain category was similar to the two pre-pregnancy BMI classification criteria (low: criterion for adolescents 40.8%, criterion for adults and 43.4%; adequate: criterion for adolescents 29.3%, criterion for adults and 29.1%; high: criterion for adolescents 29.8%, criterion for adults and 27.4%) Most adolescents showed insufficient weight gain, regardless of the pre-pregnancy BMI classification criterion. This pattern remained in the separate analyses of mothers of full- and pre-term newborns.

Pre-pregnancy BMI and weight gain showed a positive and significant association. Insufficient weight gain predominated in pregnant women classified as thin and with adequate weight under both pre-pregnancy BMI criteria. Excessive weight gain predominated in the overweight categories. The results were similar when analyzing early and late adolescents separately (Table 2).

**Table 2:** Weight gain classification according to pre-pregnancy body mass index (BMI) category, age group, and the World Health Organization (WHO) criteria to categorize pre-pregnancy BMI for adolescents and adults.

Pre-pregnancy BMI classification	Weight gain category						p-value *
	Low		Adequate		High		
	n	%	n	%	n	%	
All							
Criterion for adolescents							< 0.010
Thinness	39	41.9	35	37.6	19	20.4	
Adequate	1,853	43.2	1,311	30.6	1,123	26.2	
Overweight	471	35.1	355	26.4	517	38.5	
Obesity	144	34.4	100	23.9	174	41.6	
Criterion for adults							< 0.010
Thinness	283	40.7	257	36.9	156	22.4	
Adequate	1,842	46.0	1,176	29.4	984	24.6	
Overweight	426	38.0	282	25.1	414	36.9	
Obesity	116	36.1	74	23.1	131	40.8	
Early adolescents							
Criterion for adolescents							< 0.010
Thinness	1	100.0	0	0.0	0	0.0	
Adequate	79	32.6	107	44.2	56	23.1	
Overweight	37	26.2	46	32.6	58	41.1	
Obesity	10	28.6	4	11.4	21	60.0	
Criterion for adults							0.027
Thinness	16	29.6	26	48.1	12	22.2	
Adequate	127	43.1	107	36.3	61	20.7	
Overweight	23	39.0	15	25.4	21	35.6	
Obesity	3	27.3	1	9.1	7	63.6	
Late adolescents							
Criterion for adolescents							< 0.010
Thinness	38	41.3	35	38.0	19	20.7	
Adequate	1,774	43.9	1,204	29.8	1,067	26.4	
Overweight	434	36.1	309	25.7	459	38.2	
Obesity	134	35.0	96	25.1	153	39.9	
Criterion for adolescents							< 0.010
Thinness	267	41.6	231	36.0	144	22.4	
Adequate	1,715	46.3	1,069	28.8	923	24.9	
Overweight	403	37.9	267	25.1	393	37.0	
Obesity	113	36.5	73	23.5	124	40.0	

* Spearman’s correlation.

Note: % per row.

### Birth weight

Regardless of the classification criterion, overweight pre-pregnancy BMI was significantly associated with a higher probability of newborns with weight for gestational age > P90 and macrosomia; obesity represented a significant risk factor for weight for gestational age > P90. Using the WHO classification criterion for adolescents showed no association between thinness and birth weight. On the other hand, when the WHO criterion for adults was used, thinness was significantly associated with a higher probability of low and insufficient birth weight (Table 3).

**Table 3:** Association of birth weight of full-term newborns with pre-pregnancy body mass index (BMI) and gestational weight gain according to two pre-pregnancy BMI classification criteria.

Variable/Category	Birth weight classification											
	Weight for gestational age *						Birth weight in grams *					
	< P3 (n = 96)		< P10 (n = 309)		> P90 (n = 560)		Low (n = 211)		Insufficient (n = 1,485)		Macrosomia (n = 158)	
	OR	95%CI	OR	95%CI	OR	95%CI	OR	95%CI	OR	95%CI	OR	95%CI
Pre-pregnancy BMI classified according to WHO criterion for adolescents												
Pre-pregnancy BMI *												
Adequate	1.0	-	1.0	-	1.0	-	1.0	-	1.0	-	1.0	-
Thinness (n = 85)	2.5	0.7-8.1	1.0	0.4-2.7	0.6	0.2-1.4	2.4	1.0-5.7	1.4	0.9-2.3	0.5	0.1-3.5
Overweigh (n = 1,296)	0.8	0.5-1.3	0.7	0.5-1.0	1.5	1.2-1.8	0.6	0.4-0.8	0.7	0.6-0.8	2.1	1.5-3.0
Obesity (n = 402)	0.3	0.1-1.2	0.8	0.4-1.3	1.7	1.2-2.3	0.5	0.3-1.0	0.5	0.4-0.7	1.3	0.7-2.4
Weight gain *												
Adequate	1.0	-	1.0	-	1.0	-	1.0	-	1.0	-	1.0	-
Low (n = 2,312)	2.5	1.5-4.3	2.0	1.5-2.7	0.6	0.5-0.8	2.4	1.7-3.5	1.6	1.4-1.9	0.7	0.4-1.1
High (n = 1,781)	0.8	0.4-1.6	0.9	0.6-1.3	1.5	1.2-1.8	0.6	0.4-1.0	0.7	0.6-0.8	1.7	1.1-2.5
Pre-pregnancy BMI classified according to WHO criterion for adults												
Pre-pregnancy BMI *												
Adequate	1.0	-	1.0	-	1.0	-	1.0	-	1.0	-	1.0	-
Thinness (n = 642)	1.7	0.9-3.0	1.2	0.8-1.7	0.7	0.5-1.0	2.3	1.6-3.4	1.7	1.4-2.1	0.9	0.5-1.6
Overweight (n = 1,085)	0.8	0.4-1.4	0.8	0.6-1.1	1.5	1.2-1.8	0.5	0.3-0.8	0.8	0.6-0.9	2.2	1.5-3.2
Obesity (n = 306)	0.2	0.0-1.5	0.9	0.5-1.7	1.7	1.2-2.4	0.7	0.4-1.5	0.6	0.4-0.8	1.4	0.7-2.8
Weight gain *												
Adequate	1.0	-	1.0	-	1.0	-	1.0	-	1.0	-	1.0	-
Low (n = 2,466)	2.4	1.4-4.1	1.9	1.4-2.5	0.6	0.5-0.8	2.3	1.7-3.3	1.7	1.5-2.0	0.7	0.4-1.1
High (n = 1,639)	0.8	0.4-1.7	0.9	0.6-1.2	1.5	1.2-1.9	0.6	0.4-1.0	0.7	0.6-0.8	1.7	1.2-2.5

95%CI: 95% confidence interval; OR: odds ratio; P: percentile.

Note: adjusted by country, and maternal size and age.

* Reference group for pre-pregnancy BMI: adequate. Reference group for gestational weight gain: adequate. Reference group for birth weight: adequate weight. Reference group for weight for gestational age: between P10 and P90.

Both pre-pregnancy BMI classification criteria significantly and positively associated maternal weight gain below the recommendation with weight for gestational age < P3, < P10, low birth weight, and insufficient weight. On the other hand, excessive weight gain was significantly associated with an increased risk of macrosomia and children with weight for gestational age > P90 (Table 3). 

The adjusted linear regression for pre-term newborns showed a significant and positive association between maternal weight gain rate and birth weight.

## Discussion

This study compared two classification criteria for pre-pregnancy BMI and showed that, when compared to the adolescent-specific criterion, the adult criterion underestimated the BMI category classification in some cases. Among full-term newborns, pre-pregnancy BMI categories of overweight and obesity, and excessive maternal weight gain were associated with birth weight categories indicating excess. Conversely, pre-pregnancy thinness and maternal weight gain below the recommended levels increased the likelihood of birth weight deficits. For preterm newborns, the rate of maternal weight gain was positively associated with birth weight.

Several authors from Brazil have compared the classification of pre-pregnancy BMI according to criteria for adolescents and adults. In total, two studies ^13^ ^,^ ^14^ reported a similar level of agreement to that in this study, whereas one study ^31^ showed a higher level of agreement. When analyzing by age group, evidence suggests greater agreement among girls aged 15 years or older than with younger ones ^12^ ^,^ ^14^, which could stem from sexual maturation increasing body mass, resembling that of adults ^12^. Regarding this, the Bangdiwala chart showed that the degree of agreement between the two classification criteria progressively increased with age, until it obtained almost perfect scores for 19-year-olds for the categories of overweight and obesity.

Classifying adolescents with the adult criterion in some cases assigns a lower pre-pregnancy BMI category and generates higher weight gain recommendations ^12^ ^,^ ^14^. Some authors ^14^ ^,^ ^31^ suggest classifying the pre-pregnancy BMI of adolescent girls with the criterion designed specifically for this group by the WHO (which considers sex, age, and stage of growth), but this approach remains unclear and unstandardized. Determining the most appropriate criterion to classify pre-pregnancy BMI in adolescents is important.

Regardless of the criterion to classify pre-pregnancy BMI, this study found excess weight to be more prevalent than thinness, reflecting the shift in the nutritional profile of adolescents ^32^, in line with other Latin American studies ^12^ ^,^ ^13^ ^,^ ^31^. Pregnant women with overweight or obesity had the highest gestational weight gain ^12^, increasing the risk of postpartum weight retention ^33^ ^,^ ^34^, chronic diseases, and perpetuation of the cycle of overnutrition ^35^ ^,^ ^36^. This underscores the importance of achieving a healthy weight during postpartum, particularly before subsequent pregnancies.

Excessive pre-pregnancy BMI and excessive weight gain have been associated with weight for gestational age > P90 and macrosomia at birth ^35^ ^,^ ^36^, the latter was also found in this study and that of Estrada-Restrepo et al. ^24^ in Colombia in proportions such as low weight. Both alterations have serious implications, increasing adiposity in newborn ^37^ and the risks of developing overweight, obesity, insulin resistance, and metabolic syndrome in childhood, adolescence, and adulthood ^38^ ^,^ ^39^.

In contrast, a significant proportion of women had inadequate weight gain due to deficits ^12^, (particularly those with pre-pregnancy BMI categories of adequacy and thinness) that exacerbate nutritional deterioration. Thinness and insufficient gestational weight gain, as observed in this and other studies, are linked to lower newborn weights ^7^ ^,^ ^40^ ^,^ ^41^ ^,^ ^42^, increasing the risks of morbidity, mortality, cognitive development deficits, and chronic diseases later in life ^43^ ^,^ ^44^. These outcomes impose economic burdens on healthcare systems and negatively affect human capital development.

Low birth weight constitutes a public health indicator that has been documented as the main alteration in children of adolescents ^43^. However, this and other studies have found insufficient weight in about 25% of the newborns of Latin American women of different ages ^22^ ^,^ ^24^ ^,^ ^45^ ^,^ ^46^. Some researchers ^22^ ^,^ ^45^ ^,^ ^47^ suggest that this weight category can have deleterious effects on health (such as those of low weight), which supports the need to strengthen maternal nutritional surveillance so that all children can reach a minimum of 3,000g.

Finally, more than 60% of pregnant women in this study had weight gain outside the recommended range, consistent with findings from Mexico ^12^ and Brazil ^13^. Since adolescent weight gain is influenced by preconception nutritional status, eating habits, prenatal care quality, psychological factors, and social determinants of health and nutrition, these alarming trends underscore the urgent need for contextualized, comprehensive, and interdisciplinary prenatal care ^4^.

Adolescent girls, a priority group, must receive specific and differentiated prenatal and postnatal care to respond to the physiological, psychological, and socioeconomic needs and risks that adolescence and gestation demand ^48^ ^,^ ^49^. For this, health systems should establish guidelines that strengthen prenatal care, emphasizing adequate weight gain according to the nutritional status with which the woman begins her pregnancy and the recovery of a healthy weight in the postpartum. The results of this study, which support the relationship between maternal weight and birth weight, stress the need to evaluate the progressive weight gain during pregnancy. To this end, Restrepo et al. ^50^ and Rincón et al. ^51^ consolidated a database from which they developed charts to monitor gestational weihtg gain in Latin American adolescents to promote adequate newborn weight. 

Among the strengths of this study, it is noteworthy that this is the first to describe the performance of pre-pregnancy BMI and the weight gain of adolescent pregnant women in association with the birth weight of their children in Latin America. Also, it used a novel method, the Bangdiwala chart, to compare two pre-pregnancy BMI classification criteria. This study has limitations, including the absence of databases in some Latin American countries and the low quality of data from national systems, underscoring the need for standardized perinatal records. The reliance on secondary data influenced the quality and completeness of the dataset. While data from research projects met this study’s criteria, data from institutional or national systems showed a substantial loss, with missing variables and potential inaccuracies in weight records, introducing selection bias and limiting the generalizability of the findings. Additionally, two of the countries in this study had no preterm newborn information.

In conclusion, the pre-pregnancy BMI and maternal weight gain of adolescent girls were associated with their newborns’ birth weight. Regardless of the BMI classification criterion, the most prevalent condition was overweight, which favored excessive weight gain. This, in turn, was associated with newborns who were large for their gestational age and macrosomic. This highlights the need to focus on addressing overweight issues in adolescents. The least prevalent condition was thinness, which was associated with insufficient weight gain and, consequently, newborns with low birth weight, insufficient weight, and small size for their gestational age. To avoid inadequate recommendations for weight gain, future research should establish the best criterion to classify adolescents’ BMI and promote its consistent use by healthcare providers.

## Data Availability

The research data are available upon request to the corresponding author.

## References

[1] Organización Mundial de la Salud (2020). El embarazo en la adolescencia.

[2] Triviño-Ibarra C, Acosta Castro F, Veintimilla Cedeño J (2019). Embarazo precoz riesgos, consecuencias y prevención. Dominio de las Ciencias.

[3] Mann L, Bateson D, Black KI (2020). Teenage pregnancy. Aust J Gen Pract.

[4] Organización Panamericana de la Salud Organización Mundial de la Salud Fondo de Población de las Naciones Unidas Fondo de las Naciones Unidas para la Infancia (2018). Acelerar el progreso hacia la reducción del embarazo en la adolescencia en América Latina y el Caribe 2018.

[5] Lima RJCP, Batista RFL, Ribeiro MRC, Ribeiro CCC, Simões VMF, Lima PM (2018). Prepregnancy body mass index, gestational weight gain, and birth weight in the BRISA cohort.. Rev Saúde Pública.

[6] Trombe KSD, Rodrigues LS, Nascente LMP, Simões VMF, Batista RFL, Cavalli RC (2021). Is birth weight associated with pregestational maternal BMI BRISA Cohort, Ribeirão Preto, Brazil. Braz J Med Biol Res.

[7] Yu Z, Han S, Zhu J, Sun X, Ji C, Guo X (2013). Pre-pregnancy body mass index in relation to infant birth weight and offspring overweight/obesity a systematic review and meta-analysis. PLoS One.

[8] Vats H, Saxena R, Sachdeva MP, Walia GK, Gupta V (2021). Impact of maternal pre-pregnancy body mass index on maternal, fetal and neonatal adverse outcomes in the worldwide populations a systematic review and meta-analysis. Obes Res Clin Pract.

[9] Institute of Medicine (2009). Weight gain during pregnancy: reexamining the guidelines.

[10] World Health Organization (2006). WHO child growth standards. Length/height-for-age, weight-for-age, weight-for-length, weight-for- height and body mass index-forage: methods and development.

[11] World Health Organization (1995). Physical status: the use of and interpretation of anthropometry. Report of a WHO expert committee..

[12] Sámano R, Chico-Barba G, Martínez-Rojano H, Godínez E, Rodríguez-Ventura AL, Ávila-Koury G (2018). Pre-pregnancy body mass index classification and gestational weight gain on neonatal outcomes in adolescent mothers a follow-up study. PLoS One.

[13] Pinho-Pompeu M, Paulino DSM, Morais SS, Crubelatti MY, Pinto Silva JLE, Surita FG (2019). How to classify BMI among pregnant adolescents A prospective cohort. Public Health Nutr.

[14] Amaral J, Vasconcelos GM, Torloni MR, Fisberg M, Sampaio IPC, Guazzelli CAF (2015). Nutritional assessment of pregnant adolescents comparison of two popular classification systems. Matern Child Nutr.

[15] Fondo de las Naciones Unidas para la Infancia (2011). Estado mundial de la infancia 2011. La adolescencia, una época de oportunidades..

[16] World Health Organization BMI-for-age (5-19 years)..

[17] World Health Organization Everyday actions for better health - WHO recommendations..

[18] Villar J, Ismail LC, Victora CG, Ohuma EO, Bertino E, Altman DG (2014). International standards for newborn weight, length, and head circumference by gestational age and sex the Newborn Cross-Sectional Study of the INTERGROWTH-21st Project. Lancet.

[19] Villar J, Giuliani F, Fenton TR, Ohuma EO, Ismail LC, Kennedy SH (2016). INTERGROWTH-21st very preterm size at birth reference charts. Lancet.

[20] INTERGROWTH-21st. Sobre INTERGROWTH-21st..

[21] World Health Organization Low birth weight..

[22] Yamamoto RM, Schoeps DO, de Abreu LC, Leone C (2009). Peso insuficiente ao nascer e crescimento alcançado na idade pré-escolar, por crianças atendidas em creches filantrópicas do município de Santo André, São Paulo, Brasil. Rev Bras Saúde Mater Infant.

[23] Arrechea García GM, Castro Barberena A, Jiménez Estrada G, Gómez Fernández I, Peréz Morales A, Gómez Valdivia M (2023). Asociación entre antropometría materna y peso del neonato a término Cienfuegos, 2020-2021. MediSur.

[24] Estrada-Restrepo A, Restrepo-Mesa SL, Ceballos N, Mardones F (2016). Factores maternos relacionados con el peso al nacer de recién nacidos a término, Colombia, 2002-2011. Cad Saúde Pública.

[25] Nguyen MT, Ouzounian JG (2021). Evaluation and management of fetal macrosomia. Obstet Gynecol Clin North Am.

[26] Tribunal Internacional de Núremberg El Código de Nuremberg 1947..

[27] Asociación Médica Mundial Declaración de Helsinki de la AMM - principios éticos para las investigaciones médicas en seres humanos 2013..

[28] Borghi E, de Onis M, Garza C, Van den Broeck J, Frongillo EA, Grummer-Strawn L (2006). Construction of the World Health Organization child growth standards: selection of methods for attained growth curves.. Stat Med.

[29] Cohen J (2013). Statistical power analysis for the behavioral sciences statistical power analysis for the behavioral sciences.

[30] Bangdiwala SI, Shankar V (2013). The agreement chart. BMC Med Res Methodol.

[31] Barros DC, Saunders C, Santos MMAS, Líbera BD, Gama SGN, Leal MC (2014). The performance of various anthropometric assessment methods for predicting low birth weight in adolescent pregnant women. Rev Bras Epidemiol.

[32] Corvalán C, Garmendia ML, Jones-Smith J, Lutter CK, Miranda JJ, Pedraza LS (2017). Nutrition status of children in Latin America. Obes Rev.

[33] Groth SW, Holland ML, Kitzman H, Meng Y (2013). Gestational weight gain of pregnant African American adolescents affects body mass index 18 years later. J Obstet Gynecol Neonatal Nurs.

[34] Moll U, Olsson H, Landin-Olsson M (2017). Impact of pregestational weight and weight gain during pregnancy on long-term risk for diseases. PLoS One.

[35] Agudelo-Espitia V, Parra-Sosa BE, Restrepo-Mesa SL (2019). Factors associated with fetal macrosomia. Rev Saúde Pública.

[36] Sun Y, Shen Z, Zhan Y, Wang Y, Ma S, Zhang S (2020). Effects of pre-pregnancy body mass index and gestational weight gain on maternal and infant complications. BMC Pregnancy Childbirth.

[37] Starling AP, Brinton JT, Glueck DH, Shapiro AL, Harrod CS, Lynch AM (2014). Associations of maternal BMI and gestational weight gain with neonatal adiposity in the Healthy Start study.. Am J Clin Nutr.

[34] Gu S, An X, Fang L, Zhang X, Zhang C, Wang J (2012). Risk factors and long-term health consequences of macrosomia a prospective study in Jiangsu Province, China. J Biomed Res.

[35] Nordman H, Jääskeläinen J, Voutilainen R (2020). Birth size as a determinant of cardiometabolic risk factors in children. Horm Res Paediatr.

[40] Uchinuma H, Tsuchiya K, Sekine T, Horiuchi S, Kushima M, Otawa S (2021). Gestational body weight gain and risk of low birth weight or macrosomia in women of Japan a nationwide cohort study. Int J Obes.

[41] Aji AS, Lipoeto NI, Yusrawati Y, Malik SG, Kusmayanti NA, Susanto I (2022). Association between pre-pregnancy body mass index and gestational weight gain on pregnancy outcomes a cohort study in Indonesian pregnant women. BMC Pregnancy Childbirth.

[42] Chang WH, Lee WL, Wang PH (2021). Gestational weight gain and birth weight of newborn.. Taiwan J Obstet Gynecol.

[43] López González A (2020). Sobre los factores de riesgo del bajo peso al nacer. Rev Cuba Aliment Nutr.

[44] Robaina Castellanos GR (2017). Bajo peso al nacer, prematuridad y enfermedades crónicas en la adultez. Rev Cubana Pediatr.

[45] Costa R, Caldevilla DE, Gallo P, Figueiredo B, Leone C (2013). Incidência e características dos recém-nascidos de peso insuficiente de uma coorte de neonatos de um hospital público regional de área metropolitana. J Hum Growth Dev.

[46] Zimmer M, Oyes Ontiveros J (2020). Factores maternos asociados al peso al nacer del recién nacido en embarazadas adolescentes de Salta - Capital Argentina años 2002-2011. Rev Salud Pública Nutr.

[47] Mardones F, Acuña J (2021). Evolution of perinatal outcomes and sociodemographic variables in Chile (1996-2017). J Dev Orig Health Dis.

[48] Mann L, Bateson D, Black K (2020). Teenage pregnancy. Aust J Gen Pract.

[49] World Health Organization (2016). WHO recommendations on antenatal care for a positive pregnancy experience.

[50] Restrepo S, Rincón M, Restrepo A, Carrilho T, Kac G, Pulgarín J (2023). Gestational weight gain charts for Latin American adolescents. PLoS One.

[51] Rincón MVB, Restrepo-Mesa SL, Carrilho TRB, Kac G, Samur EA, Pulgarín JSC (2024). Establishment of a Latin American dataset to enable the construction of gestational weight gain charts for adolescents. PLoS One.

